# Virtual clinical assessment in medical education: an investigation of online conference technology

**DOI:** 10.1007/s12528-022-09313-6

**Published:** 2022-04-21

**Authors:** Harish Thampy, Sarah Collins, Elora Baishnab, Jess Grundy, Kurt Wilson, Timothy Cappelli

**Affiliations:** School of Medical Sciences, Faculty of Biology, Medicine & Health, University of Manchester, Manchester, UK

**Keywords:** Virtual assessment online, Healthcare professional education, Qualitative intervention

## Abstract

As a result of the Covid-19 pandemic, medical education institutions were suddenly and unexpectedly faced with making significant changes in delivering their clinical assessments to comply with social distancing requirements and limited access to clinical education centres. Seeking a potential solution to these new circumstances, we designed, implemented and evaluated an online virtual OSCE, as a ‘proof of concept’ intervention study. Our qualitative research involved document analysis of the stages of decision-making and consultation in designing the intervention, and thematic analysis based on the perspectives and experiences of the key stakeholders (final year students, clinical examiners, simulated patients and faculty staff who acted as station assistants), gathered through surveys with Likert-scale questions and free text comments, and online discussion groups which were recorded and transcribed. From our analysis, we identified four themes: optimising assessment design for online delivery, ensuring clinical authenticity, recognising and addressing feelings and apprehensions, and anticipating challenges through incident planning and risk mitigation. Through the data gathered at each stage of the intervention, and the involvement of key stakeholders in the design and evaluation, our study highlights examples of effective practice for future applications of online technologies in assessment, provides guidance for designing and implementing online virtual assessment, and lays a foundation for comparative, longitudinal research on the significant and increasing roles played by technology in healthcare professional education and practice.

## Introduction

Assessments of competence within clinical practice have conventionally taken place in face-to-face settings, either through standardised simulation-based examinations (at the ‘shows how’ level of Miller’s (1990) pyramid) or through workplace-based assessments in clinical settings (at the ‘does’ level of Miller’s pyramid). The Objective Structured Clinical Examination (OSCE) is a well-established example of the former, and is ubiquitously used across undergraduate and postgraduate healthcare settings to provide a standardised assessment of a learner’s clinical skills (Newble, 2004).

The OSCE was first described in the 1970s as an alternative to historic short and long-cases that posed questionable reliability and validity as ways of assessing learners’ clinical competencies (Harden et al. 1975). In an OSCE, candidates participate in a number of clinical tasks by rotating through a sequence of different stations each involving examiners, simulated or real patients, and a range of in-station resources and props, creating a high-fidelity and authentic assessment which mimic encounters experienced in real-world clinical environments. Stations typically last 5–10 min with 14–18 stations in total often used to create an entire OSCE circuit (Khan et al. 2013). Examiners remain within each OSCE station and assess every candidate who undertakes that station in turn using standardised marking rubrics (Daniels & Pugh, 2018). OSCE stations are designed to reflect everyday clinical competencies such as consultation skills (taking a history from a patient, explaining a diagnosis or treatment plan), physical examination skills, prescribing, data interpretation and clinical reasoning abilities. In blueprinting an OSCE, educators therefore need to ensure that each station’s content remains authentic to the clinical encounter they are assessing (Gormley et al. 2012). The notion of authenticity in assessment is not limited to healthcare education with an increasing drive to ensure that assessment tasks resemble meaningful performances in real world contexts (Gulikers et al. 2004).

OSCEs till date have traditionally be delivered as an in-person face-to-face assessments. However, in spring 2020, Covid-19 became a global pandemic. Healthcare professions educators were suddenly and unexpectedly faced with mandated social distancing requirements and loss of their usual teaching, learning and assessment environments due to widespread closure of institutions’ campuses and significantly restricted access to clinical settings. Whilst many programmes suspended their planned assessments for lower-stakes decision making, there remained a requirement to deliver clinical assessments in an altered form, for cohorts such as final-year medical undergraduates prior to graduation to practice and for postgraduate licensing examinations (Boursicot et al., 2020). In parallel, much of the classroom-based learning (as opposed to clinical placement learning) for clinical and communication skills moved online, with attention turned to the design and implementation of virtual online education environments. This raised questions and challenges for medical educators worldwide in how to virtually replicate, in an authentic form, the enactment of clinical skills to the standard required to become safe, competent doctors, including, for example, patient assessments, communication skills, prescribing ability and data interpretation skills.

The authors of this study are medical education leaders at a large UK medical school and were similarly faced with urgently addressing this unexpected new need to implement alternative delivery methods for our clinical competency assessments that removed the need for in-person attendance given the exigencies of the global covid pandemic. There was national General Medical Council guidance in place which asked medical schools to prioritise completion of studies for their final year medical students in order to allow them to join the clinical workforce. Fortunately, we had already conducted our usual in-person OSCEs earlier that year and thus had only a small cohort of students remaining who either had either been unable to sit or who had not passed this assessment and as such were due to take a further attempt. We therefore set out to create and deliver a novel small-scale yet high-fidelity, authentic online OSCE to assess this subset of final year medical students’ clinical competence.

To inform our work, we conducted an initial literature review (May 2020), to establish what was already known about virtual OSCE-style assessments. Yet, despite a plethora of empirical and evaluative studies exploring the implementation of face-to-face OSCE assessments, there was a noticeable paucity of literature exploring alternative ways of delivering these examinations to negate or reduce the requirement for in-person attendance. We identified only one relevant paper which evaluated participants experiences of using Skype to conduct a four station virtual OSCE (Langenau et al., 2014). However, their assessment content was limited to the single competency of clinical communication and was delivered asynchronously across an eight week testing window. As such, our endeavours to design and deliver a larger scale, multi-competency synchronous virtual assessment was conducted without the benefit of prior work to which we could link to or advance upon.

However, in the interim period from initial study delivery to final approved submission of this paper, we acknowledge that there has been a sudden rise in the number of published outputs exploring virtual/ online OSCE assessments. However, review of this new literature reveals that much of the recently published work explore virtual/ online OSCEs has been entirely descriptive in nature (Blythe et al., 2021; Craig et al., 2020; Deville et al., 2021; Donn et al., 2021; Hopwood et al., 2021; Shehata et al., 2021; Updike et al., 2021). The few papers that have adopted a more research based approach to their chosen implementation method have been limited to short-reports or evaluations of student participants only (Hannan et al., 2021; Hytönen et al., 2021; Kakadia et al., 2021; Khan et al., 2021; Savage et al., 2021).

As such, whilst the literature relating to virtual/ online OSCEs has recently expanded, questions remain regarding the views and experiences of all participants, beyond that of only student candidates. We therefore adopted a proof-of-concept research perspective to conduct a full stakeholder evaluation to investigate: What are the views and experiences of all participating stakeholders in regards to effective deployment of virtual OSCEs?

Whilst this work focuses on assessment, it was contemporaneous with moves to create online/virtual teaching and learning activities. As such, this work was conducted to also inform our understanding of how similar technological approaches could be integrated within aligned teaching and learning activities.

## Method

### Background and setting

This online OSCE was delivered to 23 final year medical students who were still required to sit their Finals examinations at a UK medical school. The OSCE is combined with written applied knowledge papers to form the high-stakes end-of-year examinations, successful completion of which allows for progression to graduation.

### Design processes

In reviewing possible assessment platforms, we created a list of key requirements that we felt were important to fulfil in order to ensure that the technology did not add a barrier to the assessment process (Table 1). Our institution had recently acquired Zoom® (Zoom Video Communications, Inc, San Jose, Calif) as their approved meeting platform with provision of professional licences to all staff. As a result, we reviewed this platform against the requirements and were satisfied that this tool was able to meet them all.

**Table 1:** Requirements of online platforms

Enables candidate, examiner and simulated patient to see and hear all parties
Allows others such as timekeepers and external examiners to observe discreetly
Offers functionality to share in-station resources such as documents, videos or images
Accommodates students securely entering a station at a given time
Houses the ability to record the session for moderation purposes
Avoids need for additional hardware aside from user’s own device
Operates within data protection / local governance requirements
Maintains examination security throughout the process

The online Zoom-delivered OSCE utilised 12 stations, each lasting 9.5 min, delivered across two consecutive days. Stations were sampled to match the blueprint of the in-person OSCE and included the following competencies:

- Consultation and reasoning skills, using simulated patients (SPs)
- Data interpretation and prescribing tasks, using in-station resources provided to candidates through the platform’s ‘share screen’ function or web links
- Practical skills needed by clinicians to examine patients, using the display of images and video montages for candidates to interpret and critique.

Each station was hosted by the examiner who was helped by a ‘station assistant’ (SA) recruited from school administrative staff. SAs served as timekeepers by verbalising timing alerts as the station progressed. In addition, they documented any incidents and actions taken to resolve them, and facilitated communication between station participants and the central examination team as required.

Examiners, timekeepers and simulated patients (SPs) all remained within the same station link for the duration of the OSCE. Candidates rotated through each station in turn, as they followed their schedule of allocated station Zoom links. Students were held in a waiting room until the examiner was ready to admit each student in turn. All stations were automatically recorded using cloud storage.

Invigilation involved examiners confirming candidates’ identity against photocards and requesting a 360-degree camera room sweep at the start of each station to ensure that candidates were taking the assessment alone. Candidates’ performance was marked using an online marksheet identical to the tablet-based marking system used in previous in-person examinations, and with which the examiners were already familiar.

Each OSCE station was scheduled within 20-min intervals to allow any technical issues to be resolved. Post-exam data analysis and reporting were conducted in line with our usual processes.

In the weeks leading up to the assessment itself a number of training and familiarisation exercises were conducted for all participants (students, SPs, examiners and SAs) given how novel the online approach was for all concerned. This included live Q&A sessions, production of guidance documents, mock run-throughs and specifically for candidates, additional clinical skills training specific to remote consultations.

### Participants and recruitment

This intervention involved 23 final year students, 23 clinical examiners, 32 SPs and 28 SAs. The student candidates involved the entire subgroup of final year students who were still to undertake their final clinical examinations. Although all students had previously undertaken conventional in-person face-to-face OSCEs in earlier years of the undergraduate medical programme, this was their first online OSCE experience. Whilst participation in the online OSCE was mandatory in order to meet institutional and external regulatory requirements for graduation, student involvement in the subsequent evaluation was entirely voluntary.

The 23 examiners were purposefully recruited from a wider bank of examiners that regularly examine in our OSCEs and following email invites, were selected based on their long-term experience of examining in medical school OSCE assessments. None of the authors of this work served as examiners to prevent conflicts of interest and preserve the integrity of the findings. Similarly, SPs who had high level of expertise and experience in simulated assessments were approached to participate in this assessment. Lastly, the 28 SAs were convenience sampled from medical school staff primarily based on their availability to participate on the given assessment dates.

### Evaluative instruments and approaches

We took a ‘proof-of-concept’ approach in our design and analysis of this assessment intervention that was in keeping with the interpretivist ontology inherent within qualitative research studies. This entailed: (a) enlisting different forms of expertise at each stage in the process, (b) gathering data through evaluations of the experience by the various parties involved, (c) documenting our decision-making processes as a multidisciplinary team.

To answer the research question, we utilised both document analysis and post-assessment evaluation involving all participants. The former drew upon extensive documentation produced throughout the design and implementation phases and included minutes from planning groups, standard operating procedures, notes from training sessions, guidance documents and on-the day ‘field notes’ from external examiners (experienced assessment experts from another institution, invited to observe the process). The post-assessment evaluation was conducted using two methods. Firstly, an electronic survey form was distributed to all participating students and examiners, utilising Likert-scale responses and free-text fields. 18/23 of students and 32/38 examiners responded. Secondly, online discussion groups were held to which all participating SPs and SAs were invited. Four discussion groups with SPs (32/32 attended) and two discussion groups with SAs (13/28 attended) were conducted, ranging from 18–32 min in length.

### Analytic approaches

All documents data were analysed for views and insights from the different experts, and for the stages in the decision-making process that informed the design and delivery of the intervention, with events and points of interest collated into themes using combined content and thematic document analyses principles as described by Glenn (2009). After initial superficial reading and subsequent thorough in-depth reading, documentary content was analysed for key and/or recurring themes that were then categorised into our three underpinning research questions. In regards to the survey, all numerical data and free-text responses were entered into spreadsheets. Numerical responses were analysed for descriptive summary statistics whilst free-text comments were deductively analysed for recurring themes against each research question. Lastly, discussion groups were all recorded and transcribed verbatim with identifiers removed to preserve anonymity of participants. Then, taking each group of participants (medical students, clinician examiners, SPs, SAs) in turn, the data were coded by two of the authors to identify recurring themes and sub-themes (Braun & Clarke, 2006). The different participants and players contribute different perspectives that together provide rich insights into the enablers and challenges relating to the online assessment approach. Thus, some themes relate to certain groups of participants but not to others.

### Ethics

In line with the University of Manchester research ethics committee guidelines, this project did not require formal ethics approval. As an education evaluation, it involved participants in their medical school roles as students, educators, members of staff and simulated patients at the University of Manchester. For the online discussions, consent was sought from all participants for the discussion to be recorded. All participants in both the online surveys and discussion groups also consented for anonymised data to be used in later research outputs. All data were fully anonymised. No individual’s identity or personal information was revealed or used as part of the process of data collation and analysis. All recordings were deleted following transcription or note-taking.

## Results

Four distinct analytic themes were generated from the data, each representing key considerations for assessment design and delivery. Each theme is illustrated below utilising quotes from the qualitative data and numerical scores from survey responses.

### Optimising station design for online delivery

This first theme relates to the processes of decision-making and conversations between colleagues, through which existing stations contained with the assessment bank were selected and adapted for online delivery. For example, consultation-based stations with SPs were conducted online to assess history taking, reasoning, communication and sharing information skills. Stations assessing data interpretation and prescribing skills were conducted through shared screen functionality or web links containing in-station resources. The virtual format of the OSCEs precluded certain skill areas being assessed, such as touch-based physical examination. These gaps were addressed through incorporating audio clips, images and video montages requiring students to interpret abnormal findings in virtual examination stations.

Data from the document analyses reveal the deliberations of the assessment team in considering the impact that image sizing and resolution may have on students’ ability to view clinical images or data sheets sufficiently. Similarly, attention was paid to anticipated issues when using in-station video recordings. Concerns were raised about the ability of the conference platform to share a playing video file smoothly, given bandwidth connectivity, and so instead a decision was taken to have these files accessible from a webpage that students could play from their own device.

Although all but one examiner agreed that their station had been suitably designed for online delivery, the unfamiliarity of these station adaptations posed concerns for students in advance of taking the assessments. 15 of the 18 student respondents expressed concerns, particularly in relation to the potential for technical issues to disturb their assessment experience:

“Prior to the OSCE, I was concerned that the examination videos would be too long or hard to see signs in”. **Student 3**

Despite the majority of stations functioning well with these technical adaptations, there remained a few instances where this had not worked as well as planned:

“Whilst good that links to photos were used, instead of screen sharing which would have been more difficult in terms of internet connection, the prescription for review and the information for the colleague discussion station were unnecessarily difficult to navigate”. **Student 5**

### Ensuring clinical authenticity

This second theme relates to whether and how the online virtual assessment was relevant to, and/or exhibited a closeness of fit with, clinical practice and students’ clinical competence. The assessment intervention was delivered towards the start of the Covid-19 pandemic and as such, remote consultations remained at that time a relatively unfamiliar approach, not only for students but healthcare workers generally. The impact of this change, and the challenges presented for the students in their consultation approach, was noted:

As this was the first time there was an online OSCE, and not all doctors are experienced in virtual clinics etc, it felt like there would be a larger variety of experience and expectation of candidates' online consulting skills compared to the well-established expectations of face-to-face OSCEs. It is therefore hard to feel as confident as for a face-to-face OSCE. **Student 8**

Students’ worries particularly focussed on the potential reduction in non-verbal communication cues due to the online format. However, despite such concerns, 17 of the 18 student respondents agreed or strongly agreed that they found they were able to communicate effectively with their SP. The shift to virtual consultations was viewed by some to offer both immediate and future benefit to students’ own clinical practice.

It effectively tested the student's powers of observation (not examination) and also allowed for an assessment of their verbal communication via technology. **Examiner 4**

It felt like we had learned a lot of skills in terms of being able to conduct consultations on video. **Student 18**

There were differences, similarities and adaptations in ways of using language and communicating, and in enacting clinical skills, in the virtual online format as compared to face-to-face. With regard to students’ expressions of empathy and rapport, simulated patients in all discussion groups were in agreement that it was possible to pick up and respond to these in the online environment:

I was a bit concerned wondering how empathetic they were going to be, not being face-to-face. And at first I had a couple that didn’t seem that empathetic, but lo and behold, the next two were fine. **SP 4**

You could just sense the ones that were listening to you or the ones like maybe you said they’d just have their notes and were just going to do it robotically, this is the way that it’s got to be done, rather than those that come off piste and just, be them. And, you know, they’re listening. You can tell as well. **SP 18**

The online environment did, however, require simulated patients to make adaptations in order to maximise their responsiveness and their communication:

Through the course of the OSCEs, I found that there was a certain kind of subconscious Zoom technique that I’d started to apply by the end of them. Where, at the beginning I was quite – naturalistic as I would do in a normal OSCE. And then I found that, actually for Zoom you have to talk in complete sentences. And make it really obvious when you’ve stopped talking. **SP 16**

You can still do a lot of things, or pick up on a lot of things, through [the online format]. You still can, read the situation, and you have to use your, hearing, more I guess because you can’t see their whole body, you can’t pick up on their body language, but you can certainly hear. **SP 3**

In relation to nonverbal communication and eye contact, a variety of differences and challenges compared to face-to-face were observed by students and examiners. For example:

Suddenly changing to the online format was quite scary. Especially given how important body language and building a rapport is for OSCEs. **Student 9**

The station was in a hospital setting with an acutely ill patient. So the realism was affected since the SP was in her normal clothes sat on her sofa. **Examiner 8**

Simulated patients observed how direction and focus of gaze were altered, and the effects this had on the communication:

[Online], your face is full on, whereas in real life, you look away more, you might be able to look to the side, and rarely is everybody looking directly at each other, in a real head-on way. Which feels quite confident, but also could be slightly aggressive because you’re just staring right at someone. And because you’ve got a full frontal picture of someone, if someone feels uncomfortable, you can tell quite quickly, because they start looking down and looking away, and the minute someone turns, you can take inference from that, quite quickly I think. It’s like there’s nowhere to hide. Like the spotlight’s on you. **SP 17**

Lastly in this theme, students, examiners and SPs recounted their observations, strategies and adaptations, to express emotions and feelings on screen (in the case of examiners, to conceal emotions and to remain neutral).

The way people come across on camera/digitally is substantially different from in person, and that could impact the whole feeling of a station and global scoring. I noticed that whilst in real life, different examiners come across differently in trying to hold a neutral face for example, online we can see them directly the whole time, so it may be worth making them aware to not be off-putting unknowingly. **Student 6**

I tried to zone it out really. And I know they couldn’t see me, but part of me had in my head I didn’t want them to be put off. **SA 4**

### Recognising and addressing feelings and apprehensions

This third theme addresses the ways in which participants’ feelings and apprehensions about the assessment intervention were recognised and addressed. The survey results show that the use of the Zoom platform to deliver this assessment posed prior concern for only 3 of the 18 student respondents, compared with 20 of the 32 examiner respondents. Feedback from both groups of participants revealed the importance of prior training, live Q&A sessions and guidance documents, in easing anxieties and building confidence prior to the OSCE. All students particularly valued the written guidance they received in advance of the assessment that detailed technical requirements, assessment expectations and incident mitigation approaches. Examiner data similarly supports the benefit of detailed prior information, with the online Q&A session and mock run-throughs proving especially valuable for this group of participants.

Overall, the lead-up in webinars, the guidance document specifically, the two mock stations, and the support of the staff, was pretty good. **Student 13**

In such uncertain times, I greatly valued the guidance document and appreciate the extra information given compared to normal face-to-face OSCEs. Although it may not have made a huge difference in candidate performance, it made us feel substantially more confident and capable and settled a fair bit of anxiety. **Student 1**

For prescribing, doing the mock OSCEs and practicing prior to the final really helped alleviate any concerns I had. As in effect I was able to have a trial run before the final OSCEs. **Examiner 17**

The beneficial effect of these student familiarisation strategies was clearly visible to other station participants:

The thing that really came across to me was the students actually seemed very very relaxed. And very confident, during the whole process. Far easier and more relaxed than I’ve ever noticed [face-to-face]. **SP 10**

I feel like the students generally looked more settled, more comfortable – comfortable in their own space I guess – in a comfortable familiar safe place – and that’s incredibly comforting. So I think you could see how different they looked to an ordinary OSCE environment – to an ordinary OSCE situation. **SP 23**

They were very calm. I think they seemed more relaxed. Almost as though, they were proper doctors. **SA 11**

Despite unfamiliarity with the virtual assessment experience, students reported a number of unique benefits to online delivery, with particular high-scoring agreement relating to the comfort and convenience of taking an assessment in their own homes and the removal of travel to examination centres. However, whilst the majority of examiners valued the convenience of remote assessments, several reported the additional prior preparation and on-the-day tasks required of this new role:

I would still prefer to have face to face contact. It is quicker and more convenient to be able to ask questions/clarify concerns when face to face. **Examiner 24**

There is far more preparation for examiners to do in the online OSCE compared to face to face where the centres have prepped everything for the examiner to just turn up. **Examiner 11**

Perhaps convenient is not the correct word, but making judgements on clinical skills via Zoom is sub-optimal compared to face to face where a more holistic view of the candidate can be appreciated. **Examiner 27**

There was recognition among the students that the intervention, and the commitment from everyone involved to making it a success, had a positive impact on the student experience of the assessment:

Whilst staff at examinations always say that they're on our side in wanting us to do well, it sometimes feels and they sometimes look like they're not. However, this time I did trust the staff and believe in the system by the end. This was probably due to smaller candidate size and therefore more personal webinar Q&As etc. This does positively impact candidate confidence and acceptance of the circumstances. **Student 15**

### Anticipating challenges: incident planning and risk mitigation

This fourth theme addresses the ways in which the assessment team put in place plans to manage unexpected incidents, and strategies adopted to mitigate risk. Whilst no significant incidents were reported, 9 of the 32 examiner respondents reported minor technical issues occurring in their station, all of which were promptly resolved to the students’ satisfaction. Interestingly, responses to this same question differ in the student data, with 14 of the 18 student respondents reporting a minor technical issue arising in their station, and only 2 students reporting that they did not feel this was resolved to their satisfaction.

There was overwhelming agreement from all parties that the detailed incident management strategy in place successfully reduced pre-assessment worries, although concerns remained until successful completion of the entire assessment:

Whilst guidance was given regarding unexpected incidents and I knew exactly who to contact, there was still some anxiety about unexpected incidents, because I didn't know what to expect having not examined like this before and because I wasn't physically in the same place as the organising team. I recognise this is unfounded really, because it is possibly easier to call someone for advice than it is to find someone on an OSCE circuit. **Examiner 20**

[My] only concern regarding technical problems was that my wi-fi would get interrupted and if this were to happen, how it would affect the examiner's perception and also myself when starting the station again. **Student 7**

I was concerned that less obvious internet connection issues would not be taken into account, e.g. frequent freezes which the examiner may not see, missing patient cues due to this or responding slowly/less appropriately due to connection or timing problems. **Student 11**

[I was] worried about my IT competence or technical glitches impacting on the students' high stakes exam performance and/or mark. **Examiner 14**

Examiners reported particular concerns in the run up to the assessment regarding their ability to simultaneously keep time, manage station technical requirements and make robust assessment decisions with provision of rich feedback. The introduction of station assistants into the plans were particularly appreciated in alleviating these worries:

The volunteer was definitely really helpful. She also did the posting of information on the chat for the student. Without this I had a bit of cognitive overload when trying to juggle timing, chat, recording and actual examining. **Examiner 22**

The inclusion of SAs however, introduced challenges in ensuring that their role as time-keeper and station facilitator did not serve as a distraction to the candidate:

I don’t even know if they thought I was a human - I was just a noise! **SA 6**

There were moments where I felt like saying anything got in the way. It felt better to interrupt the SP than the student! **SA 9**

The additional time allowed between students brought respite and enabled all tasks to be completed smoothly:

It was clear that 20 minutes leeway per station was helpful and appropriate for dealing with connection problems. It seemed to account for obvious problems like a candidate not being able to enter a station or dropping out midway through - however I was still worried about less obvious ones. **Examiner 6**

The 10-minute gap between each student, I thought that was good, because it gave a bit of breathing space, and also it was good I thought for the examiner, to get all the information. **SP 12**

When issues did arise, participants described ways these were overcome, sometimes with the effect of consolidating the relationship of trust and support between the participants in their station:

On the second day I had to use my personal hotspot, as I explained this in each station the examiners and SPs were very patient and understanding to help me get through the stations. **Student 15**

I had one student and there was a technical overlap, so you know he’d ask something, you’d go to answer and then you’re constantly overlapping a little but it didn’t matter – just like normal conversation really so it didn’t matter. **SP 26**

For the purposes of trouble-shooting and providing support during the days of the assessment, messaging groups were set up, so that, as one participant put it, everyone could ‘bounce messages off each other”. These routes of communication were warmly welcomed:

SP 13: I thought the ‘whatsapp’ group was actually brilliant because we were all supporting each other

Other SPs in the group: ((collective agreement)).

SP 14: Yeh we don’t have that normally and actually that felt great to be able to just – to have that. **SPs in discussion**

We said, if there was one thing to come out of this, messaging groups are such an easy thing to set up **SA 12**

Lastly in this theme, it became evident that whilst online OSCEs reduced some of the significant resource demands inherent with in-person OSCEs (for example physical space, timetabling issues and physical equipment), they introduced new resource requirements. For example, additional funding was required, to provide examiners with upgraded ‘professional’ accounts on the selected platform allowing additional hosting and recording functionality. Furthermore, the additional online training and familiarisation exercises for all participants also increased the up-front resourcing required.

### Summary of analytic themes

As illustrated in the data examples above, the themes – optimising for online delivery, ensuring clinical authenticity, recognising and addressing feelings, and anticipating challenges–encompassed a range of perspectives and experiences from all stakeholders involved. Taken together, these themes provide a series of key elements to consider when planning, implementing and evaluating an online technological intervention.

## Discussion

Through exploration of the different stakeholder perspectives, our study shows the potential for online virtual assessment interventions which combine the expertise and recognise the contributions of the various participants, to achieve successful implementation. The experiences and evaluations from the students, examiners, SPs and SAs show that, for the most part, these key players positively valued the approaches adopted, with a number of areas highlighted for future onward development.

### Technological affordances, assessment adaptations and limitations

In making decisions about the initial choice of online delivery platform, it became apparent that there was a lack of suitable web-based tools to conduct synchronous cyber assessment (Chao et al., 2012), with online assessment mainly confined to asynchronous assessment (online, open-book examinations) or knowledge-based synchronous assessments with incorporated virtual invigilation/proctoring. Indeed, at the time of our work, we were unable to identify any dedicated platform for delivering virtual OSCEs. Instead, previous work had utilised existing online conferencing systems such as Langenau et al.’s (2014) use of Skype® to deliver online OSCEs. The authors, however, highlight this tool’s limited functionality and that over half of students reported technical difficulties.

Our decision to use Zoom®, given its accessibility, functionality and ability to simulate the rotational format of OSCE stations, was supported in the evaluation data. As reported above, students were much more comfortable using this tool in comparison to examiners likely reflecting their prior familiarity in its use as part of preceding teaching activities. Recent short reports describing adaptations during the Covid-19 pandemic have similarly used this platform to successfully deliver remote clinical assessments (Craig et al., 2020; Hannon et al., 2020; Lara et al., 2020).

Although we successfully modified stations for online delivery, the inability to assess touch-based physical examination remains a key drawback to the virtual approach, which learners can find concerning (Novack et al., 2002). Similar to Philips et al. (2020), we instead utilised multimedia to simulate such encounters, though the absence of ‘bedside cues’ and the digitalised nature of the encounter could have the potential to adversely affect the authenticity due to differences from previous clinical experiences. Despite students fearing in advance that the online format would negatively impact their consultation approach, this proved unfounded with the majority of students and SPs positively commenting on their continued ability to consult effectively in the virtual environment. However, it was noted that remote online consultations introduced particular communication and technical skills distinct from in-person OSCEs. As suggested by our data, and supported by Prettyman et al. (2018), non-verbal dimensions can be more difficult to assess in the online environment, although facial expression is emphasised through the headshot afforded via screens. The absence of certain non-verbal cues, such as body posture, can heighten awareness and responsiveness to other linguistic and paralinguistic features: word choices, pace and tone of voice, facial expression, shows of maintaining eye contact, and animation in movement of the upper body and hands. These aspects can more closely attune the examiner who is observing (and the simulated patient who is responding to the candidate) to the candidate’s communication performance.

### Training needs for examiners, students and SPs

Given the constraints (and opportunities) that technology provides for observing and communicating when assessing clinical practice online, it is essential that these are clearly specified and described in detail in order to provide bespoke, relevant training for all concerned. For example, in examiner training, and SP calibration, the differences in all dimensions of communicating online need to be identified and explored, and any corresponding adjustments or exceptions need to be incorporated into the process of observing, interacting with, and marking the students. Furthermore, students should be provided with opportunities to practice and develop their online consultation skills prior to being expected to successfully demonstrate these in online assessments. Sartori et al. (2019) describe how senior candidates undertaking a telemedicine OSCE struggled in basic clinical tasks such as eliciting a full history and making treatment plans. They suggest that the virtual environment introduced an obstacle in patient assessment and care. These concerns were addressed through delivering training in remote consultation skills with opportunity for clinical exposure to this area of practice prior to OSCE-based assessment (Cantone et al., 2019). Remote consultation skills training has also been used successfully even for sensitive clinical encounters, such as breaking bad news (Daetwyler et al., 2010).

‘Assessment literacy’ describes candidates' understanding of why certain assessment strategies and tools are used in the way they are, what the assessment criteria are, and how and why particular standards are set (O'Donovan et al., 2008). As illustrated in our findings in relation to feelings and apprehensions, taking part in an online OSCE was novel and unfamiliar to many of our participants. We were concerned that this could potentially trigger higher anxiety levels for the student candidates, risking negative performance impact. Therefore, promoting students’ assessment literacy became a key aim. Our post-OSCE evaluation highlighted the importance students paid to the strategies we used to seek to address this including e-mail updates, question and answer sessions and practice station run-throughs.

Examiners similarly expressed prior concern about the technical aspects to assessing online. As part of examiner recruitment, we therefore additionally established each participant’s comfort with technology and familiarity with the selected platform to help target additional training requirements where needed. Our evaluation research has highlighted the value in producing a comprehensive technology instruction manual, similar to that reported by Langenau and colleagues (Langenau et al., 2014). Our manual described the overall assessment process and specific expected responsibilities and tasks to be completed for pre- during- and post-OSCE stages. It was supplemented with webinar-based training sessions, in which participants could discuss concerns, resolve queries and trial the platform’s functionality, further building confidence.

Our findings revealed examiner concerns relating to the perceived additional work required of them, both in terms of further training and preparation and on-the-day roles and responsibilities, in comparison to in-person OSCEs. Particular concern focused on their ability to simultaneously manage the technical elements of a station whilst making complex assessor judgements. We were able to alleviate some of these challenges through the inclusion of station assistants. In doing so, we were also able to award the station assistants a significant role in the assessment process: a role that drew on their expertise and responsibilities as administrators and student support staff. From the perspective of SPs, they were more comfortable with online delivery of their role given their experience of online auditions in their work as actors, and so their training instead focussed on the unique format elements to the online assessment approach. SPs valued the opportunity for prior online calibration of their role, allowing familiarisation of the scripts with the additional option of demonstration videos, to standardise the level of emotion to be played without the distractions of process-related queries. Just as for all stakeholder groups, SP involvement in the evaluation of the intervention highlighted examples of good practice for future applications of online technologies in teaching, learning and assessment.

### Implementing measures for incident planning and risk mitigation

As shown in our study, despite rigorous planning and testing, there remained the potential for unexpected issues arising on the day of OSCE delivery. Building confidence in the overall process for all participants, through careful detailed contingency planning for potential incidents, was essential for successful execution of this novel assessment. These plans were clearly communicated in advance, with details of how and when to enact contingency measures, and when further escalation should occur. Table 2 summarises examples of potential actions that can be taken for anticipated incidents.

**Table 2:** Incident / contingency planning

Incident Type	Incident	Response
System	Limited / unstable internet connectivity	Testing prior to assessment. Consider provision of internet access dongles
	WiFi dropouts	Ethernet cable connection to be used where possible
	Bandwidth overload	Limiting other demands on the connection through closing unnecessary applications
	On-the-day loss of connection/ platform problems	Candidates, examiners and simulated patients all provided with emergency phone number for live technical support Additional timed spacing between stations to allow for catch-up of minor interruptions Creation of reserve stations to allow candidates who suffered significant outages to complete the station at the end of cycle
	In-station resource issues e.g. problems with shared-screen viewing or web link access	Creating alternative back-ups for use if needed, e.g. identical resource to be hosted on a webpage if shared-screen does not work and vice versa
People	Examiners or SPs unexpectedly unavailable on the day of the OSCE	Maintain contact details for both examiners and SPs to allow for on-the-day communication Recruit reserve examiners and SPs who attend training and are familiar with allocated reserve stations scripts
	Third party interruptions	Requiring candidates, examiners and SPs to make arrangements in advance to manage potential disruptions (e.g. childcare, phones on silent)
	Candidate fails to arrive at station	Having on-the-day contact information for all candidates. Creating examiner communication groups (e.g. on a mobile app) to quickly cascade information across a cycle relating to candidates (or stations)

Common to many of the incident responses, is the importance of creating clear routes of communication for all participants. Candidates, examiners and SPs were given emergency contact information to access technical assistance. Likewise, the OSCE administration team were provided with contact details for candidates, examiners and SPs in event of investigating unexpected absences. Mobile-platform communication apps offered an easy and effective team communication route that facilitated distant discussions between examiners and SPs with the central administration team and our data demonstrates their use was strongly appreciated. Our evaluation revealed that all incidents that arose were system-based rather than people-based and were all fortunately minor and quickly resolved. There were no incidents or challenges that arose that we had not accounted for in advance.

Our findings demonstrate that we successfully delivered this new online clinical assessment approach without any significant incidents arising. However, there were conflicting accounts between students and examiners about how frequently minor incidents arose, such as temporary loss of connection or brief audio-visual interruptions. Whilst 9 of the 32 examiner respondents reported such incidents occurring, this was at odds with 14 of the 18 student respondents reporting the same. This may suggest that students were experiencing minor issues that were not reported or made apparent to the examiner, though without detriment to their assessment experience. Elucidating the nature of these unrecognised issues is needed to help minimise recurrence in future delivery.

### Benefits and challenges for online virtual assessment going forwards

As illustrated in our study, stakeholders perceived a variety of benefits and drawbacks to virtual OSCE assessments. The convenience of remote assessments was appreciated more by students than examiners, with the latter group expressing potential concern about the impact this novel approach may have had in making assessment decisions. Whilst concerns may remain about the use of screen-based assessor judgements on candidate’s performance versus in-person direct observation, remote scoring has been shown to be as reliable as on-site scoring (Chen et al., 2019). Examiners should be flexible and understanding of the unfamiliar assessment format, and candidates need to have confidence that the format does not jeopardise their marking.

For future, the online format carries additional logistical benefits. For example, recording of stations can allow for the use of asynchronous marking, helping alleviate any examiner recruitment concerns (Bautista & Manalastas, 2017). Allowing examiners to assess remotely can also remove some of the logistical barriers than may limit their attendance in conventional in-person examinations. Furthermore, the planning and delivery phases can be easily designed to ensure that the assessment remains inclusive to all candidates, with reasonable adjustments applied to remove barriers that may substantially disadvantage an individual on grounds of disability GMC (2019). We found, for example, that we were just as able modify station timing to accommodate additional time requests as would be applied during in-person assessments. Secondly, although it was not required, we had planned to modify the display of text for in-station resources to meet the needs of students with specific learning difficulties alongside creating audio-visual elements with consideration for those with hearing or visual impairment through the provision of closed captioning.

Online assessments can also introduce potential equity of access issues for candidates who may not have the connectivity and device resources required, and provision of these may need to be managed by the institution. Furthermore, students may find the remote OSCEs anxiety-provoking or may experience upset or distress as a result of station content (Marshall & Jones, 2003). As a future modification, we have appreciated that it is important to build pastoral support mechanisms into remote delivery, to replicate face-to-face support students would seek from staff in these instances: such as the creation of a ‘virtual support room’ for students to access if they require.

### Implications for future

Although the recent implementation of remote online clinical assessments has largely been undertaken through force and not choice given the impact of the Covid-19 pandemic, our successful introduction of this approach into the assessment armamentarium has identified a number of potential ongoing advantages that may reshape future clinical assessment delivery across both undergraduate and postgraduate settings. We recognise however that this proof of concept study was conducted on a small cohort of students and at a single institution. Further work is therefore needed prior to larger-scale implementation, exploring themes across different medical schools and Higher Education institutions.

In terms of future research in this area, the data collection methods we chose to employ were relevant, practical and achievable. Our methods were aligned not only to the research question but to the various group participants involved. For example, it was possible to convene time slots to suit SPs and SAs to meet together for discussion online, while the asynchronous form of the online survey enabled busy clinicians and final year students to contribute to the evaluation in their own time. Furthermore, the richness of the insights and perspectives on the use of technology gleaned through these methods demonstrate their potential for a wider-scale study to assess the impact of such interventions across student cohorts and healthcare professional education programmes.

Our study suggests that virtual online assessments are not only capable of assessing the core clinical skills of communication and social interaction, but indeed may also bring advantages and enhancements. For example, SPs and SAs commented on the calm demeanour of the students online (also observed in studies of patients’ experiences (Greenhalgh et al., 2016), and examiners commented on the ease with which hitches can be reported online compared to face-to-face. These experiences point to possibilities for online technologies to enhance performance and the ability to assess (for examiners) and respond to (for SPs) healthcare professional students’ performance.

To conclude, remote online assessments offer a viable option through which to deliver clinical assessments in the future. Our proof of concept intervention study has shown that online clinical assessments are feasible, hold potential to bring about resource and economy savings, enable the maintenance of social distancing requirements within the context of the current pandemic, and allow for many clinical competencies to be assessed in an as-robust a way as conventional OSCEs. Most significantly perhaps, their ongoing implementation reflects a newly recognised importance of remote clinical care.

## References

[CR1] Bautista JMD, Manalastas REC (2017). using video recording in evaluating students’ clinical skills. Medical Science Educator.

[CR2] Blythe J, Patel NSA, Spiring W, Easton G, Evans D, Meskevicius-Sadler E, Noshib H, Gordon H (2021). Undertaking a high stakes virtual OSCE (“VOSCE”) during Covid-19. BMC Medical Education.

[CR3] Boursicot K, Kemp S, Ong T, Wijaya L, Goh S, Freeman K, Curran I (2020). Conducting a high-stakes OSCE in a COVID-19 environment. MedEdPublish.

[CR4] Bowen Glenn A (2009). Document analysis as a qualitative research method. Qualitative Research Journal.

[CR5] Braun V, Clarke V (2006). Using thematic analysis in psychology. Qualitative Research in Psychology.

[CR6] Cantone RE, Palmer R, Dodson LG, Biagioli FE (2019). Insomnia telemedicine OSCE (TeleOSCE): a simulated standardized patient video-visit case for clerkship students. MedEdPORTAL.

[CR7] Chao K-J, Hung I-C, Chen N-S (2012). On the design of online synchronous assessments in a synchronous cyber classroom. Journal of Computer Assisted Learning.

[CR8] Chen T-C, Lin M-C, Chiang Y-C, Monrouxe L, Chien S-J (2019). Remote and onsite scoring of OSCEs using generalisability theory: a three-year cohort study. Medical Teacher.

[CR9] Craig C, Kasana N, Modi A (2020). Virtual OSCE delivery: the way of the future?. Medical Education;n/a(n/a).

[CR10] Daetwyler CJ, Cohen DG, Gracely E, Novack DH (2010). eLearning to enhance physician patient communication: a pilot test of doc com and WebEncounter in teaching bad news delivery. Medical teacher.

[CR11] Daniels VJ, Pugh D (2018). Twelve tips for developing an OSCE that measures what you want. Medical Teacher.

[CR12] Deville RL, Fellers CM, Howard ML (2021). Lessons learned pivoting to a virtual OSCE: pharmacy faculty and student perspectives. Currents in Pharmacy Teaching and Learning.

[CR13] Donn J, Scott JA, Binnie V, Bell A (2021). A pilot of a virtual objective structured clinical examination in dental education a response to COVID-19. European Journal of Dental Education.

[CR14] GMC. (2019). *Welcomed and valued: Supporting disabled learners in medical education and training*. Retrieved 16/07/2020 from

[CR15] Gormley G, Sterling M, Menary A, McKeown G (2012). Keeping it real! Enhancing realism in standardised patient OSCE stations. The Clinical Teacher.

[CR16] Greenhalgh T, Vijayaraghavan S, Wherton J, Shaw S, Byrne E, Campbell-Richards D, Bhattacharya S, Hanson P, Ramoutar S, Gutteridge C, Hodkinson I, Collard A, Morris J (2016). Virtual online consultations: advantages and limitations (VOCAL) study. British Medical Journal Open.

[CR17] Gulikers JTM, Bastiaens TJ, Kirschner PA (2004). A five-dimensional framework for authentic assessment. Educational Technology Research and Development.

[CR19] Hannan TA, Umar SY, Rob Z, Choudhury RR (2021). Designing and running an online objective structured clinical examination (OSCE) on zoom: a peer-led example. Medical Teacher.

[CR20] Hannon P, Lappe K, Griffin C, Roussel D, Colbert-Getz J (2020). An objective structured clinical examination: from examination room to Zoom breakout room. Medical Education.

[CR18] Harden RM, Stevenson M, Downie WW, Wilson GM (1975). Assessment of clinical competence using objective structured examination. British Medical Journal.

[CR21] Hopwood J, Myers G, Sturrock A (2021). Twelve tips for conducting a virtual OSCE. Medical Teacher.

[CR22] Hytönen H, Näpänkangas R, Karaharju-Suvanto T, Eväsoja T, Kallio A, Kokkari A, Tuononen T, Lahti S (2021). Modification of national OSCE due to COVID-19–Implementation and students’ feedback. European Journal of Dental Education.

[CR23] Kakadia R, Chen E, Ohyama H (2021). Implementing an online OSCE during the COVID-19 pandemic. Journal of Dental Education.

[CR24] Khan FA, Williams M, Napolitano CA (2021). Resident education during Covid-19, virtual mock OSCE's via zoom: A pilot program. Journal of Clinical Anesthesia.

[CR25] Langenau E, Kachur E, Horber D (2014). Web-based objective structured clinical examination with remote standardized patients and skype: resident experience. Patient Education and Counseling.

[CR26] Lara S, Foster CW, Hawks M, Montgomery M (2020). Remote assessment of clinical skills during COVID-19: a virtual, high-stakes, summative pediatric objective structured clinical examination. Academic Pediatrics.

[CR27] Marshall G, Jones N (2003). A pilot study into anxiety induced by various assessment methods. Radiography.

[CR28] Miller GE (1990). The assessment of clinical skills/competence/performance. Academic Medicine.

[CR29] Newble D (2004). Techniques for measuring clinical competence: objective structured clinical examinations. Medical Education.

[CR30] Novack DH, Cohen D, Peitzman SJ, Beadenkopf S, Gracely E, Morris J (2002). A pilot test of WebOSCE: a system for assessing trainees' clinical skills via teleconference. Medical Teacher.

[CR31] O'Donovan B, Price M, Rust C (2008). Developing student understanding of assessment standards: a nested hierarchy of approaches. Teaching in Higher Education.

[CR32] Phillips TA, Munn AC, George TP (2020). Assessing the impact of telehealth objective structured clinical examinations in graduate nursing education. Nurse Educator.

[CR33] Prettyman AV, Knight EP, Allison TE (2018). Objective structured clinical examination from virtually anywhere!. The Journal for Nurse Practitioners.

[CR34] Sartori DJ, Olsen S, Weinshel E, Zabar SR (2019). Preparing trainees for telemedicine: a virtual OSCE pilot. Medical Education.

[CR35] Savage A, Minshew LM, Anksorus HN, McLaughlin JE (2021). Remote OSCE experience: what first year pharmacy students liked, learned, and suggested for future implementations. Pharmacy.

[CR36] Shehata MH, Kumar AP, Arekat MR, Alsenbesy M, Mohammed Ansari A, Atwa H, Ahmed SA, Deifalla A (2021). A toolbox for conducting an online OSCE. The clinical teacher.

[CR37] Updike WH, Cowart K, Woodyard JL, Serag-Bolos E, Taylor JR, Curtis SD (2021). Protecting the integrity of the virtual objective structured clinical examination. American Journal of Pharmaceutical Education.

